# Deficiency of *Mkrn2* causes abnormal spermiogenesis and spermiation, and impairs male fertility

**DOI:** 10.1038/srep39318

**Published:** 2016-12-23

**Authors:** Xu Qian, Lin Wang, Bo Zheng, Zhu-Mei Shi, Xin Ge, Cheng-Fei Jiang, Ying-Chen Qian, Dong-Mei Li, Wei Li, Xue Liu, Yu Yin, Ji-Tai Zheng, Hua Shen, Min Wang, Xue-Jiang Guo, Jun He, Marie Lin, Ling-Zhi Liu, Jia-Hao Sha, Bing-Hua Jiang

**Affiliations:** 1 State Key Laboratory of Reproductive Medicine,Nanjing Medical University, Nanjing, Jiangsu 210029, China; 2 Department of Pathology, and Cancer Center, Nanjing Medical University, Nanjing, Jiangsu 210029, China; 3 Institute of Medical and Pharmaceutical Sciences, Zhengzhou University, Henan 450052, China; 4 Department of Neurosurgery, The First Affiliated Hospital of Nanjing Medical University, Nanjing, Jiangsu 210029, China; 5 Department of Oncology, The First Affiliated Hospital of Nanjing Medical University, Nanjing, Jiangsu 210029, China; 6 Center for Molecular Carcinogenesis, Department of Pathology, Anatomy and Cell Biology, Thomas Jefferson University, Philadelphia, PA 19107, USA; 7 Biomedical Engineer Research Center, Kunming Medical University, Kunming, Yunnan 65000, China

## Abstract

Although recent studies have shed insights on some of the potential causes of male infertility, new underlining molecular mechanisms still remain to be elucidated. *Makorin-2 (Mkrn2*) is an evolutionarily conserved gene whose biological functions are not fully known. We developed an *Mrkn2* knockout mouse model to study the role of this gene, and found that deletion of *Mkrn2* in mice led to male infertility. *Mkrn2* knockout mice produced abnormal sperms characterized by low number, poor motility, and aberrant morphology. Disruption of *Mkrn2* also caused failure of sperm release (spermiation failure) and misarrangement of ectoplasmic specialization (ES) in testes, thus impairing spermiogenesis and spermiation. To understand the molecular mechanism, we found that expression of Odf2, a vital protein in spermatogenesis, was significantly decreased. In addition, we found that expression levels of Odf2 were decreased in *Mkrn2* knockout mice. We also found that *MKRN2* was prominently expressed in the sperm of normal men, but was significantly reduced in infertile men. This result indicates that our finding is clinically relevant. The results of our study provided insights into a new mechanism of male infertility caused by the MKRN2 downregulation.

Infertility is a severe health problem leading to tremendous social consequences. Approximately 10–15% of all couples experience fertility-related problems during their childbearing years^1^ and around 50% of such cases are caused by male infertility^2^. Sperm carries paternal genetic information delivering heredity to offspring. Spermatogenesis can be divided into three stages: mitosis of spermatogonia, meiosis of spermatocytes, and spermiogenesis^3^,^4^,^5^. Although many genes, including more than 20 male germ cell-specific genes, have been identified to be essential for spermatogenesis^6^, the underlying mechanisms of spermatogenesis remain largely unknown.

The makorin (*MKRN*) gene family encodes putative ribonucleoproteins with a distinctive array of zinc finger domains^7^. Makorin-1 (*MKRN1*) is the most extensively studied member of this family of proteins^7^,^8^,^9^. Makorin-2 (*MKRN2*), formerly designated as *HSPC070*, is a new member of the Makorin family, which was first identified in human CD34+ hematopoietic stem/progenitor cells^10^. MKRN2 is a zinc finger protein containing the typical C_3_HC_4_ protein-protein interaction motif (termed the RING domain) found in most E3 ubiquitin ligases^11^,^12^. It is ubiquitously expressed in various tissues and cell lines^10^,^13^, but MKRN2 function is not known yet in mammals. We previously demonstrated that forced expression of *Mkrn2* in *Xenopus* produced impaired tadpoles with dorso-posterior deficiencies and small-head/short-tail phenotype, whereas knockdown of *Mkrn2* by morpholino antisense oligonucleotides induced a double-axis phenotype during neurogenesis^14^; however, further studies are required to fully determine the biological function of Mkrn2 in mammals.

To investigate the function of the *MKRN2* gene, we generated *Mkrn2*-knockout mice. Based on the results of our previous study, we expected that deletion of *Mkrn2* in mice may result indeficiency in the development of neurogenesis^14^. Surprisingly, *Mkrn2-*knockout mice were viable with detectable abnormal neurogenesis, but the male mice were sterile. As mice and human share similar process of spermiogenesis and spermiation^15^, these infertile *Mkrn2-*knockout mice could serve as an *in vivo* model to investigate the biological function of MKRN2 in human spermiogenesis and spermiation.

## Results

### Expression pattern of *Mkrn2* in mouse testes

**Figure 1:**
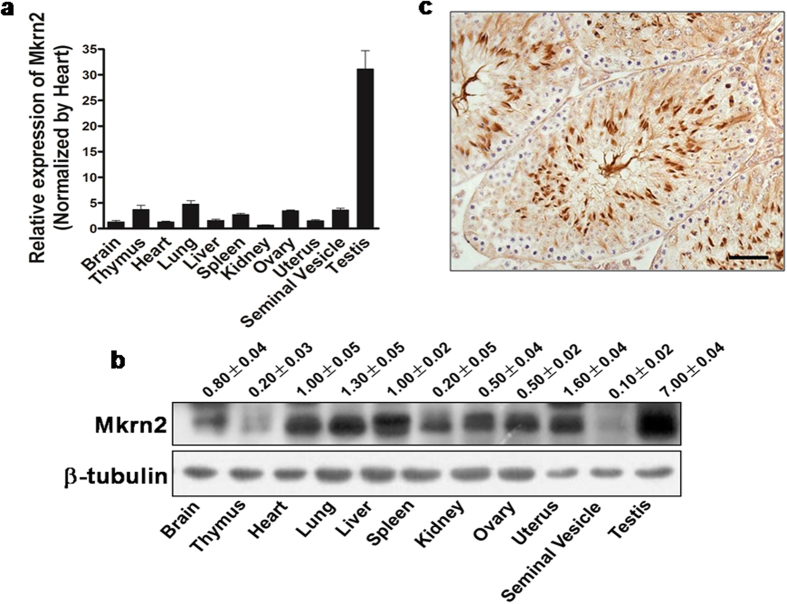
Expression profile of Mkrn2 in mice tissues. (**a**) Mkrn2 mRNA levels in different tissues were analyzed by RT-qPCR and normalized to those in liver tissue. Data in the figure are represented as means ± SE. from four mice. (**b**) The Mkrn2 protein levels in mouse organs were detected by immnuoblotting. β-Tubulin was used as a loading control. The blotting density was normalized to that of the liver. (**c**) The location of Mkrn2 inthe testis of 4-week mice was determined using immunohistochemistry. Scale bar = 20 μm.

Mkrn2 mRNA and protein expression levels were evaluated using qRT-PCR and immunoblotting analysis, respectively, in different tissue samples of mice. The *Mkrn2* mRNA levels were low in the brain, thymus, heart, lung, liver, spleen, kidney, ovary, uterus, and seminal vesicle; but were very high in the testis with 30-fold higher than those in the brain and heart tissues (Fig. 1a). Similarly, Mkrn2 protein was ubiquitously expressed at low levels in different tissues, but preferentially expressed in the testis (Fig. 1b), indicating that Mkrn2 may play a vital role in testes. Furthermore, immunohistochemical analysis revealed that Mkrn2 protein was mainly localized in the Sertoli cells and spermatids (Fig. 1c). These data implied that Mkrn2 may play an important role in spermiogenesis and spermiation.

### MKRN2 expression in infertile human sperms

**Figure 2:**
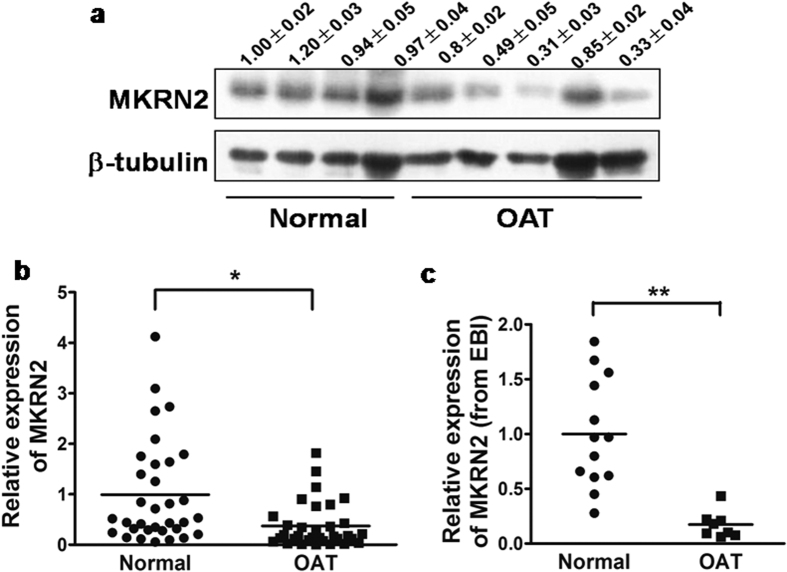
Expression of MKRN2 in human sperm. (**a**) MKRN2 protein expression levels from human sperm samples were subjected to immunoblotting analysis. (**b**) The MKRN2 mRNA levels from human sperm samples were measured by RT-qPCR. (**c**) MKRN2 mRNA expression levels of sperm samples from the US cohort were compared to available raw data extracted from the European Bioinformatics Institute (EBI) database. OAT, oligoasthenoteratozoospermia. * And ** significant difference at *P* < 0.05 and *P* < 0.01, respectively (two-tailed Student’s *t* test).

MKRN2 protein levels were lower in human sperm samples from patients of oligoasthenoteratozoospermia (OAT) than in the normal samples (Fig. 2a, Supplementary Figure S1). Similarly, the mRNA levels of *MKRN2* in the OAT patient sperms were significantly lower by more than two-fold than those in the normal group (Fig. 2b). To validate this finding in an independent cohort, we obtained MKRN2 expression data from sperm samples (including 8 OAT patients and 13 normal subjects) of American populations which was submitted to the database of European Bioinformatics Institute (EBI) by Platts and colleagues (E-GEOD-6872)^16^. By analyzing the raw data, we found that the *MKRN2* mRNA levels were significantly decreased by 5-fold in the OAT group than in the corresponding normal group (Fig. 2c). These two independent cohorts demonstrated that low MKRN2 expression levels in sperm are strongly correlated with human male infertility.

### Generation of *Mkrn2* knockout mice

**Figure 3:**
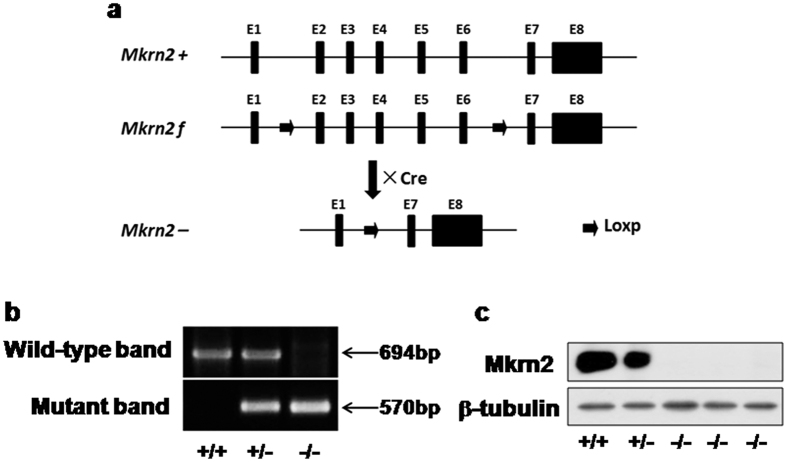
Generation of *Mkrn2* knockout mice. (**a**) Strategy for generating *Mkrn2* knockout mice. *Mkrn2*-floxed mice whose Exons (numbered black boxes) 2–6 of the *Mkrn2* gene were flanked by loxP sites (arrow) were crossed with *EIIa-Cre* transgenic mice to generate *Mkrn2* knockout mice. E, exon; f, Floxed; +, wild-type; −, mutant. (**b**) *Mkrn2* gene disruption was confirmed by PCR genotyping. Tail genomic DNA was amplified with specific primers for the wild-type (694 bp) and mutant (570 bp) *Mkrn2* alleles. (**c**) Immunoblotting analysis showing the absence of *Mkrn2* in the protein extracts of *Mkrn2* knockout testes. β-Tubulin expression was used as an internal control to indicate equal amount of loading proteins. +/+, wild-type; +/−, heterozygous; −/−, homozygous (knockout).

Model animals are very useful tools to study the functions and mechanisms of target genes^17^. To better investigate the role of *Mkrn2* gene *in vivo*, we generated *Mkrn2* knockout mice using a Cre-LoxP system (Fig. 3a). The *Mkrn2* gene in mice contains eight exons, in which the functional domains are mainly encoded by Exons 2–6. Thus, we disrupted *Mkrn2* gene by deleting these exons and introducing frameshift mutations in Exons 7 and 8, thus obtaining maximum knockout effect. The mutant allele could be transmitted from both *Mkrn2* heterozygous male and female mice, and homozygous mice could be obtained by interbreeding these heterozygous mice. *Mkrn2* heterozygous and homozygous offspring were identified using a PCR-based genotyping assay (Fig. 3b). Immnuoblotting analysis using testis extracts verified the absence of Mkrn2 protein in *Mkrn2* knockout mice (Fig. 3c). Amino acid sequence alignment showed that the MKRN2 protein in mice and human shared 88% identity (Supplementary Figure S2), suggesting that *Mkrn2* knockout mice may be useful to explore the biological function of MKRN2 in human.

### Characteristics of *Mkrn2* knockout mice

**Figure 4:**
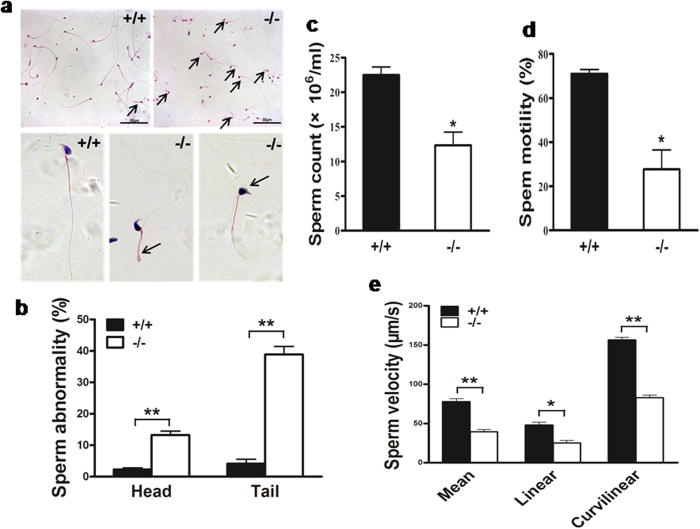
*Mkrn2* knockout mouse sperm structure and characteristics. (**a**) Sperm obtained from the cauda epididymis were stained by hematoxylin and eosin (H&E). Sperm from *Mkrn2* knockout mice were deformed: the sperm tails bent forward in the midpiece and the heads were round and irregular (arrow). Scale bar = 50 μm. (**b**) The proportions of abnormal sperm were calculated from five randomly selected microscopic fields in a double-blinded manner. Data are represented as the means ± SE. of five random microscopic fields. The sperm count (**c**),motility (**d**), and velocity (**e**) were measured by computer-assisted sperm analysis (CASA) from six different mice of corresponding phenotypes. Data are represented as the means ± SE. of six mice. Error bars represent SE. *P < 0.05, **P < 0.01 (two-tailed Student’s *t* test).

Both male and female *Mkrn2* knockout mice were viable, and had no gross abnormalities except that their weights were lighter than wild-type counterparts (Supplementary Figure S3). *Mkrn2* knockout male mice could mate with female mice, evidenced by the existing of vaginal plugs after mating. During the four-month duration of the mating test, the average litter size of wild-type male and female mice and *Mkrn2* heterozygous male and female mice were 8.6 and 8.9 pups, respectively. However, no pups were born between *Mkrn2* knockout male mice and female mice (including wild-type, heterozygous, and knockout), indicating that *Mkrn2* knockout male mice were completely sterile (Table 1). Note that female *Mkrn2* knockout mice were fertile, but displayed reduced fecundity with an average litter size of 5.9 pups. We then determined why *Mkrn2* deletion leads to male infertility. First, the lower sperm-producing ability of male *Mkrn2* knockout mice was confirmed by examining their cauda epididymis. Although a few of *Mkrn2* knockout mice produced no sperm, most of them produced sperms, but with abnormal heads and tails (Fig. 4a).The abnormality rates in the sperm heads and tails of *Mkrn2* knockout sperm were 13.2% and 38.9%, respectively, which were remarkably higher than the corresponding rates of 2.3% and 4.1% in wild-type sperms (Fig. 4b). Computer-assisted sperm analysis (CASA) showed that sperm amount was significantly reduced (Fig. 4c), and that approximately 70% of sperms lost motile ability (Fig. 4d) in the *Mkrn2* knockout mice. Although the remaining 30% of the sperms in the *Mkrn2* knockout group were motile, their mean, linear, and curvilinear velocities were significantly lower than those sperms from the wild-type mice (Fig. 4e). The observation of high rate of abnormality and low motile ability of *Mkrn2* knockout sperms is very similar to those in the sperm samples obtained from OAT patients (Supplementary video 1 and 2). Thus, male *Mkrn2* knockout mice produced abnormal sperms with a low count, reduced motility, and deformed morphology, which are similar to the most common type of male infertility in human^18^,^19^.

### Failure of spermiation in the testes of *Mkrn2* knockout mice

**Figure 5:**
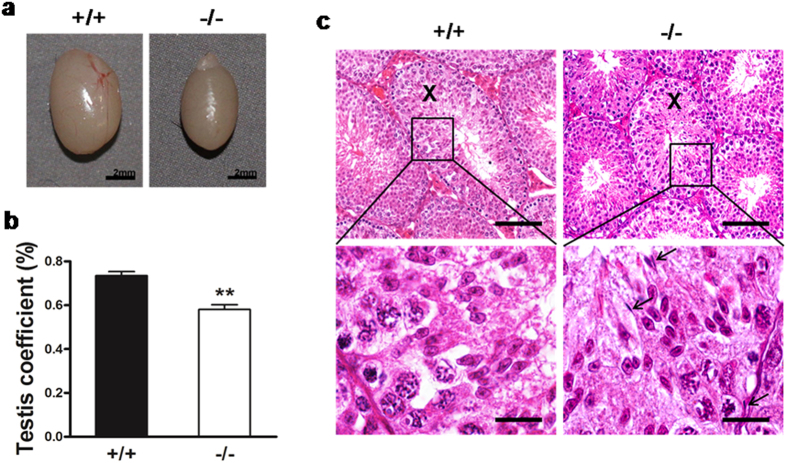
Analysis of *Mkrn2* knockout mouse testes. (**a**) Macroscopic appearance of testes. Scale bar = 2 mm. (**b**) Testis weights. The net testis weight was normalized to body weight. Data arerepresented as the means ± SE. From six testes for each phenotype. Error bars represent SE. ** *P* < 0.01 (two-tailed Student’s *t* test). (**c**) Hematoxylin& eosin (H&E) staining sections of Stage X testicular tubules. Arrows indicate the spermiation failure spermatids in *Mkrn2* knockout testes (right); these sperm should have been released at Stage X, as observed in the wild-type testes (left). Scale bar: Upper panel, 100 μm; Bottom panel, 20 μm.

The internal organs of the mice were weighed and normalized to their body weight. The relative organ weights were similar between *Mkrn2* knockout and wild-type mice in most of organs including brain, thymus, heart, lung, liver, spleen, kidney, and stomach (Supplementary Table S1); while the relative weights and volumes of testis were significantly lower in *Mkrn2* knockout mice compared to those of the wild-type counterparts (Fig. 5a,b). In the testes of *Mkrn2* knockout mice, all 12 stages of spermatogenesis were present (Supplementary Figure S4). In Stage VIII seminiferous tubules of *Mkrn2* knockout mice, all cell types can be observed, but the number of round spermatids was significantly decreased (Supplementary Figure S5a,b). And elongated spermatids (EI) were lining in the center of the tubules prior to spermiation, similar to that observed in the testis of wild-type mice (Supplementary Figure S5a). However, a distinguishable defect was found in *Mkrn2* knockout testes at Stage X (Fig. 5c). High-power magnification fields showed that *Mkrn2* knockout seminiferous tubules retained elongated spermatids (termed as spermiation failure) among germ cells, which should be released at this stage like those in the Stage X seminiferous tubules of the wild-type mice. These data indicated that Mkrn2 is important in the regulation of spermiation.

### Deletion of *Mkrn2* damages arrangement of actin filaments in the ectoplasmic specialization

**Figure 6:**
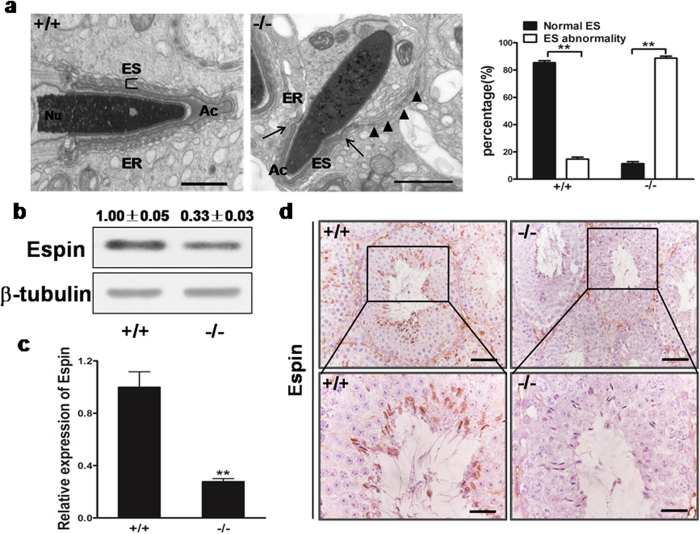
Analysis of *Mkrn2* knockout mouse ectoplasmic specialization (ES) structure. (**a**) TEM analysis of ES structure. Arrows indicate the boundary of the acrosome, and arrowheads indicate the improper arrangement of ES. Nu, nucleus; Ac, acrosome; ER, endoplasmic reticulum. Data are represented as means ± SE. From six different testis tissues for each phenotype. Error bars represent SE. **P < 0.01 (two-tailed Student’s *t* test). Scale bar = 1 μm. (**b**) Espin expression levels in the testes were analyzed by immunoblotting. β-Tubulin was used as the internal control. (**c**) The Espin mRNA levels in the testes were analyzed by RT-qPCR and normalized to that of β-Tubulin. Data are represented as means ± SE. From six different testis tissues for each phenotype. Error bars represent SE. **P < 0.01 (two-tailed Student’s *t* test). (**d**) Immunohistochemical staining of Espin in the testes shows co-localization of Espin at the apical ES and in the heads of elongated spermatids in wild-type testes (left), but no obvious staining can be observed in the tubules of the *Mkrn2* knockout testes. Scale bar: Upper panel, 50 μm; Bottom panel, 20 μm.

The ectoplasmic specialization (ES) is a specialized adhesion junction found in Sertoli cells at sites of attachment to elongated spermatids or neighboring Sertoli cells in the testes^20^. Spermiation failure can be indicative of disruption of ES^21^. We examined the ultrastructure of the testes using transmission electron microscopy (TEM). In normal testes, the ES synchronously stretches along with the acrosome, and is characterized by the presence of actin filament bundles sandwiched between endoplasmic reticulum (ER), and the two apposite plasma membranes of elongating spermid and Sertoli cells (Fig. 6a, left panel). In the *Mkrn2* knockout testes, however, the ES arrangement was disrupted, as evidenced by desynchronization with the acrosome (Fig. 6a, left and middle panels). The ES broke across off the boundary of the acrosome, and extended further, preventing proper elongation of round spermatids by Sertoli cells in *Mkrn2* knockout testes. We next examined the expression levels of the ES marker Espin. The Espin protein and mRNA levels in *Mkrn2* knockout testes were decreased to one-third of the levels detected in the wild-type group (Fig. 6b,c). Immunohistochemical analysis revealed that Espin was expressed in the region between the ES and the heads of elongating/elongated spermatids in the wild-type group, but its expression was barely detected in *Mkrn2* knockout testis (Fig. 6d). These data indicate that Mkrn2 regulates the accurate arrangement of the ES and Espin expression.

### Disruption of Mkrn2 results in sperm abnormalities

**Figure 7:**
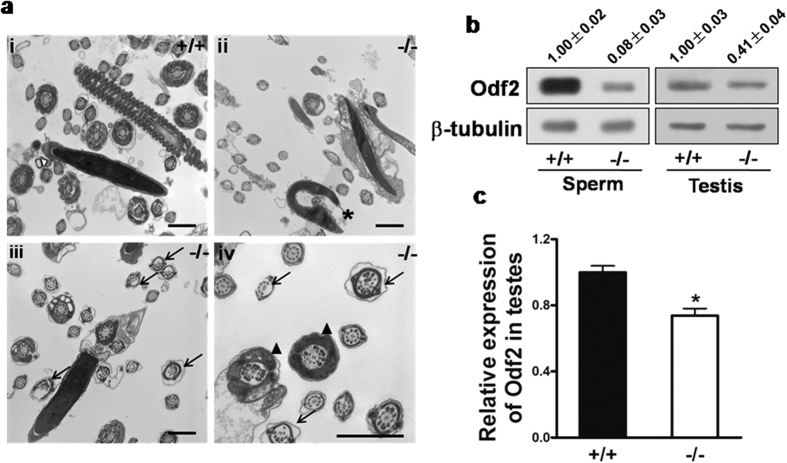
Analysis of *Mkrn2* knockout mouse sperm structures and Odf2 expression. (**a**) TEM analysis of epididymal sperm. i, Note the normal head and axoneme with typical “9 + 2” microtubule structure (nine pairs of peripheral and two central microtubules) in wild-type mice. ii–iv, Abnormalities in *Mkrn2* knockout mouse sperm were evaluated by the appearance of deformed heads (ii, asterisk), deformed “9 + 2” structures with missing and/or misarranged microtubules (iii-iv, arrows) and lack of Odf (iv, arrowhead). Scale bar = 1 μm. (**b**) The Odf2 protein levels in wild-type and *Mkrn2* knockout mouse sperm and testes, respectively. β-Tubulin was used as the loading control. (**c**) The Odf2 mRNA expression levels in the testes were determined by RT-qPCR and normalized to that of β-Tubulin. Data are represented as the means ± SE. From six different testes tissue samples for each phenotype. Error bars represent SE. *P < 0.05 (two-tailed Student’s *t* test).

To further define the nature of the abnormalities in *Mkrn2* knockout sperms, we compared the ultrastructure of sperm from the *Mkrn2* knockout and wild-type groups using TEM. Results revealed that spermatozoa were normal with well-shaped heads and tails in the wild-type mice (Fig. 7a,i). However, sperms from the *Mkrn2* knockout mice showed deformed heads, disorganized axonemal structure, and disorganized flagellar structure (Fig. 7a, ii–iv). The shapes of the sperm heads were aberrant, resulting in abnormal or missing acrosomes (Fig. 7a, ii, asterisk). Normal sperm tails contain a highly ordered structure termed the “9 + 2” axoneme^22^. The “9 + 2” axoneme arrangement in the tail flagellae of *Mkrn2*-deficient sperm exhibited two types of abnormalities (Fig. 7a, iii-iv, arrows): First, complete loss of the axoneme doublets in one side of the fibrous sheath; and second, disordered assembly of the “9+2” arrangement. These data demonstrated that Mkrn2 is required for the normal sperm development.

Next, we investigated the molecular mechanism of Mkrn2 deficiency leading to male infertility. Interestingly, we found that the outer dense fiber (Odf), which is an important component of flagellae (Fig. 7a, iv, arrowheads), was either absent or improperly arranged in the *Mkrn2* knockout epididymal sperms. This finding is consistent with the reduced expression of Odf2 protein, which is important for the regulation of sperm morphology (Fig. 7b). In addition, Odf2 mRNA and protein levels were significantly lower in *Mkrn2* knockout sperms and testes than those of the wild-type (Fig. 7b,c). This result suggest that Mkrn2 is required for both Odf2 protein and mRNA expression.

## Discussion

*Mkrn2* is a new member of the highly evolutionarily conserved makorin family, and it maybe originally created by gene duplication of *MKRN1* approximately 450 million years ago^13^. *MKRN1* plays multiple functions such as modulating gene transcription, controlling protein stabilization, and regulating tumorigenesis^23^,^24^,^25^. However, the biological function of *Mkrn2* in mammals still remains unknown. We previously found that *Xenopus mkrn2* inhibited neurogenesis during early embryonic development^14^. To further explore the *in vivo* biological function of *Mkrn2*, we generated a strain of *Mkrn2* knockout mice by using EIIa-Cre method. EIIa-Cre is widely expressed and highly efficient in gene knockout because it is expressed as early as the zygote stage^26^,^27^. Since the biological function of Mkrn2 is largely unknown, we firstly employed EIIa-Cre method to efficiently delete Mkrn2 in the whole body of mice, and to investigate the phenotype caused by Mkrn2 knockout. According to our previous study on a *Xenopus* model, we anticipated that disruption of *Mkrn2* would result in prenatal death or deficiency in neurogenesis in mice. Surprisingly, *Mkrn2* knockout mice were viable with no obvious phenotype associated with neurodeficiency. After fecundity tests, male *Mkrn2* knockout mice were found to be sterile, and no pups were born during the 4-month period of mating with female mice. This finding revealed potential role of Mkrn2 in fertility of male mice. Further analysis indicated that *Mkrn2* knockout mice either did not have sperm, or produced sperm with a low count, poor motility, and/or abnormal shapes. This condition is termed oligoasthenoteratozoospermia (OAT) in human, which is the most common cause of infertility in human. Mice are commonly used to study human diseases because of the genetic similarities between human and mice^15^,^28^,^29^. Nevertheless, the results from the animal model studies need to be confirmed in human. In this study, we showed that human MKRN2 protein and mRNA levels in the infertile OAT sperm were lower than those in the fertile sperm from a human cohort. This finding was validated by the results of the second cohort of sperm samples obtained from the EBI, in which the MKRN2 mRNA levels were downregulated in OAT sperm compared to their normospermic counterparts in the same cohort. These two independent cohorts provide direct evidence indicating that MKRN2 is significantly correlated with infertility in men. Together, the results obtained from both *Mkrn2* knockout mice and human cohorts suggest that MKRN2 is important for regulating fertility and that *Mkrn2* knockout mice may serve as a useful animal model for further studies.

To understand why disruption of Mkrn2 impairs fertility, we examined the distribution of Mkrn2 in mouse organs. While Mkrn2 was expressed at low levels in all the organs examined, it was preferentially overexpressed in the testis with up to 30-fold higher mRNA levels than those in other organ tissues. Furthermore, Mkrn2 was expressed in Sertoli cells and spermatids. Based on these results, we speculated that Mkrn2 may play an important role in testis development and in the process of spermatogenesis. As expectedly, the sizes and weights of the testes in *Mkrn2* knockout mice were lower than those in normal mouse testes. TEM analysis revealed that impaired ES structure in *Mkrn2* knockout mice^20^,^30^,^31^. ES is mainly characterized by the presence of a unique junction plaque containing parallel actin bundles sandwiched between the plasma membrane of Sertoli cells and affiliated endoplasmic reticulum^32^,^33^. ES is considered important for orienting the positions and shapes of the spermatid head and for regulating spermiation^34^,^35^, thus playing a vital role in spermiogenesis and spermiation. Histological examination revealed abnormal spermiogenesis and spermiation in the Stage X seminiferous tubules of the knockout mice, observed by retention of elongated spermatids and sloughing in the different compartments of germ cells. This is a very interesting phenomenon termed spermiation failure, which can be indicative of changes in the components of the ES^21^,^36^. Ultrastructure analyses revealed that the ES in the knockout mouse testes was desynchronized with the acrosome and stretched beyond the boundary of the acrosome. Immunoblot analysis showed that Espin was rarely detected in *Mkrn2* knockout mouse testis, indicating that the ES was severely impaired in these mice. As a specialized structure formed by Sertoli cells, ES plays a vital role in spermiogenesis and spermiation. Our results indicated that deletion of Mkrn2 in the testes, especially in Sertoli cells, impaired ES functions, thus leading to sperm abnormalities.

Except for some cases where the *Mkrn2* knockout male mice produced no sperm, most knockout mice were capable of producing lower numbers of sperm. The low sperm count maybe due to the reduced number of round spermatids in Stage VIII and/or the spermiation failure in stage X seminiferous tubules. Furthermore, deletion of the *Mkrn2* gene led to severe impairment of sperm motility, which strongly indicated abnormality in sperm tails. The same phenomenon was observed in the sperm of human patients with OAT. Sperm from the *Mkrn2* knockout group were morphologically abnormal, with deformed heads and tails bent toward the head at the principle piece. Ultrastructure analyses of cauda epididymal sperm revealed a drastically deformed nucleus and improperly arranged sperm flagellae in *Mkrn2* knockout mice. The “9 + 2” structure was partially lost or disordered. These results implied that deletion of Mkrn2 in the testes, especially in germ cells, would impair some essential components during sperm flagellar assembly. After further exploration, we found that Odf was not assembled correctly. Odf2, an important member of Odf family, has been reported to be associated with flagellar assembly^37^,^38^. The Odf2 expression levels were greatly reduced both in the sperm and testes of *Mkrn2* knockout mice, indicating that *Mkrn2* may control the expression of Odf2 directly or indirectly, consequently modulating sperm flagellar assembly. It was reported that Odf2 KO results in preimplantation lethality, suggesting that Odf2 may be an important downstream protein of MKRN2 for spermiogenesis and spermiation^39^.

This study uses an Mkrn2 knockout mouse model and two independent cohorts of human sperm samples to demonstrate the important role of Mkrn2 in male fertility. Our results demonstrate that Mkrn2 is essential for spermiogenesis and spermiation. We found that deletion of Mkrn2 in somatic Sertoli cells results in disruption of ES, leading to abnormalities in sperm heads and spermiation failure. This deletion also suppresses Odf2 expression in germ cells, disrupting the flagellar assembly of sperm tails. Furthermore, the Mkrn2 expression levels were significantly downregulated in the infertile male subjects in our study. *Mkrn2* knockout mice may serve as a model for investigating the involvement of Mkrn2 in spermiogenesis and spermiation in the future.

## Material and Methods

### Sperm sample collection and analysis

Human sperm samples have been collected for several years by the Center of Clinical Reproductive Medicine, the First Affiliated Hospital of Nanjing Medical University. Informed consents were obtained from all subjects participating in the study. Ejaculates were evaluated according to World Health Organization criteria^40^. These sperm samples and other related samples have been kept in the tissue bank of the First Affiliated Hospital of Nanjing Medical University. These samples have been prepared for cases in the First Affiliated Hospital of Nanjing Medical University biorepository, and clinical annotation is available through a database using caBIG. Cases will be classified and selected based on diagnosis using the CoPath Anatomic Pathology system as well as caBIG. No information regulated by The Health Insurance Portability and Accountability Act was included in the study, which qualifies for the status of NIH Exemption # 4. The sperm samples based on the database containing 33 infertile men diagnosed as having oligoasthenoteratozoospermia (OAT) and 33 fertile men were used for this study, and the results are showed in Supplementary Figure S1.

### Ethics statement

All animal experiments were approved by the Committee of Laboratory Animal Experimentation of Nanjing Medical University. All methods were performed in accordance with the relevant guidelines and regulations. The mice used in this study were housed in a controlled specific pathogen-free (SPF) environment and cared according to the approved protocol.

### Generation of *Mkrn2* knockout mice

To generate *Mkrn2* floxed mice, a targeting vector containing *Mkrn2* exons 2–6 flanked by Loxp sites was linearized by digestion with *Not*I was introduced into embryonic stem cells derived from mice with a 129P2/OlaHsd background by electroporation. Southern blotting analysis was used to confirm the resulting cells harboring floxed alleles of *Mkrn2*. The targeted embryonic stem cell clones were microinjected into C57BL/6 J blastocysts of female mice, which gave birth to chimeric mice. The offspring were subsequently crossed with C57BL/6 J mice to generate F1 *Mkrn2*-floxed offspring.

To generate *Mkrn2* knockout mice, *Mkrn2*-*floxed* mice that had been backcrossed with C57BL/6 J for more than five generations were crossed with transgenic *EIIa-cre* mice with a C57BL/6 J background (Jackson Laboratory) to obtain mosaic mice with the *Mkrn2*^+/*f*/−^*·EIIa-cre* genotype. The resulting mice were crossed with C57BL/6 J to obtain *Mkrn2* heterozygotes. *Mkrn2* knockout mice were generated by sibmating of heterozygotes. Genomic DNA isolated from the tails was genotyped with oligonucleotides listed in Supplementary Table S2, yielding 694-bp (wild-type) and 570-bp (mutant) products.

### Morphological and immunohistochemical analysis

The testes and epididymis tissues were dissected, fixed in Bouin’s fixatives overnight, and embedded in paraffin. Sections were stained by hematoxylin and eosin (H&E) for histological examination or immunohistochemically using antibodies against MKRN2 (Novus Biologicals) and Espin (BD Biosciences). The epididymis sections were dissected, incubated in PBS, and epididymal sperm morphology was analyzed by observing the sections on a slide with methanol fixation and H&E staining.

### Sperm analysis

Sperm motility and count were measured using a CASA system as described previously^41^. Mature sperm were isolated from the cauda epididymis dissected from sexually mature mice. The tissue was incised at four sites and placed in 0.5 mL of PBS for 15 min in a 37 °C incubator. Debris was removed, and 20 μL aliquots of sperm were placed in an 80-μm-deep chamber for the assessment of sperm motility, and the sperm count was obtained by CASA using an IVOS Sperm Analyzer (Hamilton Thorne Biosciences).

### Ultrastructure analysis

For electron microscopy, testicular and cauda epididymal sperm from adult mice were fixed in 5% (v/v) and 2.5% (v/v) glutaraldehyde at 4 °C, respectively. The specimens were embedded according to standard procedures, sectioned, and examined with a JEM-1010 transmission electron microscope (JEOL).

### RNA analysis

Total RNAs were prepared using TRIzol reagent (Invitrogen) according to the manufacturer’s instructions. First-strand cDNAs were synthesized and subjected to real-time RT-PCR analysis (Vazyme). The relative amounts of target genes were calculated by the comparative threshold cycle (Ct) method. β-Tubulin mRNA levels were used as the internal control. The oligonucleotide primer sequences used here are listed in Supplemental Table 2.

### Immunoblotting

Tissues were homogenized in ice-cold RIPA buffer supplemented with protease inhibitor cocktail (Sigma). The cell suspensions were centrifuged for 15 minutes at 15,000 rpm at 4 °C. The sperm samples were collected and homogenized in lysis buffer containing 7 M urea (Thermal Scientific), 2 M thiourea (Sigma-Aldrich), 4% (w/v) CHAPS (Sigma-Aldrich), 65 mM dithiothreitol (SunShine Biotechnology), and 1% (v/v) Protease Inhibitor Cocktail for sperm protein analysis, as described in a previous study^42^. Briefly, homogenates were subjected to four repetitions of ultrasound every 15 minutes, and then centrifuged for 1 h at 15,000 rpm at 4 °C. The extracted proteins were subjected to immnuoblot analysis for detecting MKRN2 (Novus Biologicals), Espin or Odf2 (BD Biosciences). β-Tubulin (Cell Signaling Technology) was used as the loading control.

### Statistical analysis

Data were presented as the means ± SE. of at least three independent experiments unless otherwise indicated. The Student’s unpaired *t* test was used for comparison of the wild-type and knockout mice. Values were considered significantly different at *P* < 0.05.

## Additional Information

**How to cite this article**: Qian, X. *et al*. Deficiency of *Mkrn2* causes abnormal spermiogenesis and spermiation, and impairs male fertility. *Sci. Rep.* **6**, 39318; doi: 10.1038/srep39318 (2016).

**Publisher's note:** Springer Nature remains neutral with regard to jurisdictional claims in published maps and institutional affiliations.

## Supplementary Material

Supplementary Information

Supplementary Video 1

Supplementary Video 2

## Figures and Tables

**Table 1:** Reproductive Defects in *Mkrn2* Knockout Mice.

Male (n)	Female (n)	Litter number	Pups	Litter Size (mean ± SD)
+/+ (3)	+/+ (6)	11	95	8.6 ± 1.8
+/− (11)	+/− (20)	40	354	8.9 ± 1.5
+/+, +/− (7)	−/− (7)	9	53	5.9 ± 1.3
−/− (17)	+/+ (7)	0		0
	+/− (5)	0		0
	−/− (11)	0		0

The mean litter size was calculated by dividing the number of live pups born by the number of litters. The results are from the mating of mice less than 6 months of age.
