# Supplementary material for: Deficiency of Mkrn2 causes abnormal spermiogenesis and spermiation, and impairs male fertility
Source: Sci Rep. 2016 Dec 23;6:39318. doi: 10.1038/srep39318 (PMC5180214; doi:10.1038/srep39318)

**Supplementary Information**

**Deficiency of *Mkrn2* causes abnormal spermiogenesis**

**And spermiation, and impairs male fertility**

Xu Qian^1,2,8^, Lin Wang^3,8^,Bo Zheng^1,8^, Zhu-Mei Shi^1,2,4^, Xin Ge^1,2^, Cheng-Fei Jiang^1,2^, Ying-Chen Qian^1,2^, Dong-Mei Li^1,2^,Wei Li^1,2^, Xue Liu^1,2^,Yu Yin^1,2^, Ji-Tai Zheng^1,2^, Hua Shen^5^, Min Wang^1,2^, Xue-Jiang Guo^1^, Jun He^6^, Marie Lin^7^, Ling-Zhi Liu^6^, Jia-Hao Sha^1,*^& Bing-Hua Jiang^1,2,6*^

^1^State Key Laboratory of Reproductive Medicine,Nanjing Medical University, Nanjing,Jiangsu 210029, China.^2^Department of Pathology, and Cancer Center, Nanjing Medical University, Nanjing, Jiangsu 210029, China. ^3^Institute of Medicine, University of Zhengzhou, Henan Province. ^4^Department of Neurosurgery, The First Affiliated Hospital of Nanjing Medical University, Nanjing, Jiangsu 210029, China. ^5^Department of Oncology, The First Affiliated Hospital of Nanjing Medical University, Nanjing, Jiangsu 210029, China. ^6^Department of Pathology, Anatomy and Cell Biology, Thomas Jefferson University, Philadelphia, PA 19107, USA. ^7^Biomedical Engineer Research Center, Kunming Medical University, Kunming, Yunnan65000, China.

^8^These authors contributed equally to this work.

*Corresponding authors: BH Jiang, email:[binghjiang@yahoo.com](mailto:binghjiang@yahoo.com) and [bhjiang@jefferson.edu](mailto:bhjiang@jefferson.edu); JH Sha, email: shajh@njmu.edu.cn

**Supplementary Methods**

**Diff-quik staining**

Morphology of human sperm were evaluated using the Diff-Quik staining kit (Fisher Scientific). Smears were prepared using 10 µL of sperm suspension dragged with a cover slip and allowed to air-dry. Slides were immersed in each solution of the staining kit according to manufacturer’s instructions, air-dried, and observed under a bright-field microscope[^1^](#_ENREF_1).

**Supplementary Figure Legends**

**Supplementary Figure 1. Diff-Quik staining of human sperm**

Human ejaculates were rapidly stained by Diff-Quik staining, and photomicrographed. Bar = 10 μm. The arrows indicate deformed sperm tails, and the triangle indicates a deformed sperm head.

**Supplementary Figure 2. Amino acid sequence alignment of human and mouse Mkrn2**

Pairwise alignment of human MKRN2 (GenBank accession number: AAG30426.1) and mouse Mkrn2 (GenBank accession number AAH25547.1). Of the 416 MKRN2 residues, 367 (88%) are identical between humans and mice. Identical and similar residues are highlighted in red and blue, respectively.

**Supplementary Figure 3. Body weights of mice**

Mice were weighed every 2 days after birth. Body weights of the *Mkrn2* knockout mice (-/-) were lower than those of their wild-type counterparts (+/+). Data are presented as means ± SE. from 10 mice for each phenotype. * *P* < 0.05 (two-tailed Student’s *t* test).

**Supplementary Figure 4. Hematoxylin and eosin-stained sections of Stage I–XII testicular tubules**

Mouse testes were sectioned and stained by hematoxylin and eosin. The arrows in Stage X (bottom panel) indicate spermiation failure in *Mkrn2* knockout testes; these spermatids should have been released at this stage as seen in the wild-type testes (top panel). Scale bar = 10 μm.

**Supplementary Figure 5.** **Morphology and germ cell components of Stage VIII tubules in wild-type and *Mkrn2* knockout testes**

(**a**) Hematoxylin eosin-stained Stage VIII tubules. The elongated spermatids (El) were arranged in the luminal side of Stage VIII seminiferous tubules both in the wild-type and *Mkrn2* knockout testes. (**b**) Germ cell component in Stage VIII tubules. Numbers of germ cells (spermatocytes and round spermatids, respectively) in Stage VIII tubules were counted and averaged from five random microscopic fields with 200 × magnification. Data are represented as means ± SE. * *P* = 0.0024 (two-tailed Student’s *t* test). Scale bar = 50 μm.

**Supplementary Video Legends**

The observation of high rate of abnormality and low motile ability of *Mkrn2* knockout sperm is very similar to those in the sperm samples obtained from OAT patients (Supplementary video 1 and 2).

**Supplementary Table 1. Physical characteristics of mice**

| **Parameters** | ***Mkrn2^+/+^*** | ***Mkrn2^−/−^*** |
| --- | --- | --- |
| Body weight (g)^A^ | 26.0 ± 1.7 | 23.8 ± 1.4 |
| Brain weight (%) | 1.94 ± 0.26 | 1.98 ± 0.29 |
| Thymus weight (%) | 0.24 ± 0.04 | 0.23 ± 0.05 |
| Heart weight (%) | 0.73 ± 0.09 | 0.76 ± 0.08 |
| Lung weight (%) | 0.63 ± 0.08 | 0.60 ± 0.04 |
| Liver weight (%) | 5.05 ± 0.51 | 4.94 ± 0.85 |
| Spleen weight (%) | 0.32 ± 0.08 | 0.32 ± 0.03 |
| Kidney weight (%) | 1.41 ± 0.07 | 1.46 ± 0.12 |
| Stomach weight (%) | 0.69 ± 0.08 | 0.68 ± 0.02 |

Values are presented as the means ± SD. Physical characteristics were obtained from six male mice of each genotype aged approximately 2 months. Organ weights are represented as a pecentage relative to the body weight. ^A^*P* < 0.05 (two-tailed Student’s *t* test).

**Supplementary Table 2. Primers** used in this study

| **Primer name** | **Sequence 5'-3'** |
| --- | --- |
| ***Genotyping*** |  |
| KO-F1 | TGGACAGGTGAAACGGCAAGGAAA |
| KO-R1 | AACCACAGTCGTGGCAGCCTTG |
| KO-F2 | CCTTCTGGCTCTTACCTCCT |
| KO-R2 | GCTCTTTGCCTTATTCACTTTAC |
| ***qRT-PCR*** |  |
| Espin-F | GCCAGAAAACCTGGGACAAAG |
| Espin-R | TGAAAGATTTGGTGCTGGGTAT |
| Odf2-F | GCACTGCAAAGAGGATTCCG |
| Odf2-R | GCCTTATGCTGGTTCCCAC |

**Supplementary Reference**

1 Mota, P. C. & Ramalho-Santos, J. Comparison between different markers for sperm quality in the cat: Diff-Quik as a simple optical technique to assess changes in the DNA of feline epididymal sperm. *Theriogenology* **65**, 1360-1375, doi:10.1016/j.theriogenology.2005.08.016 (2006).


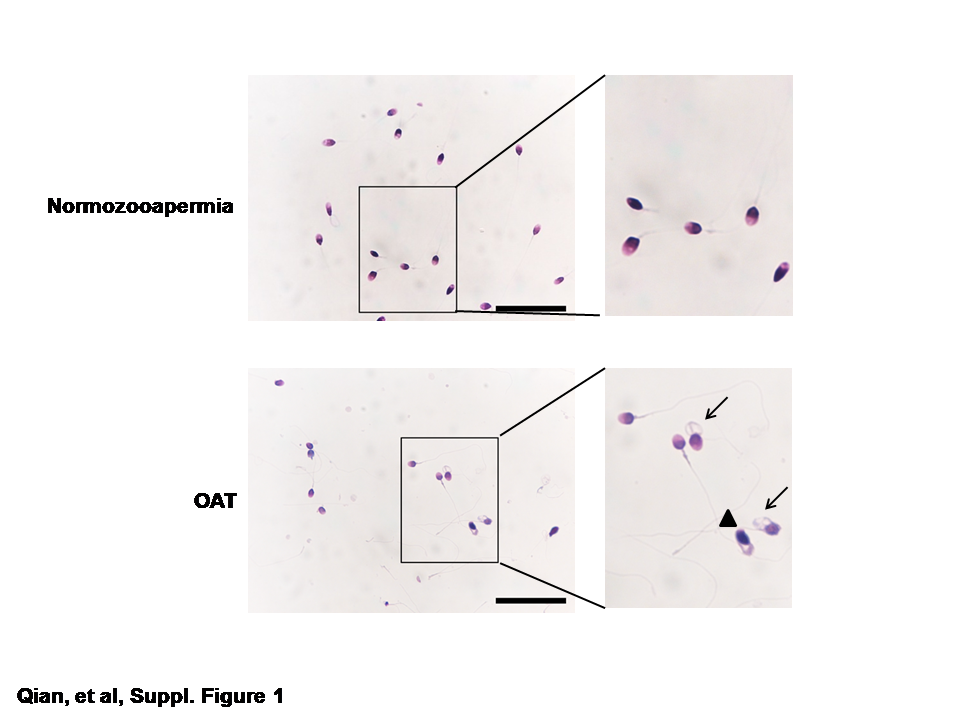

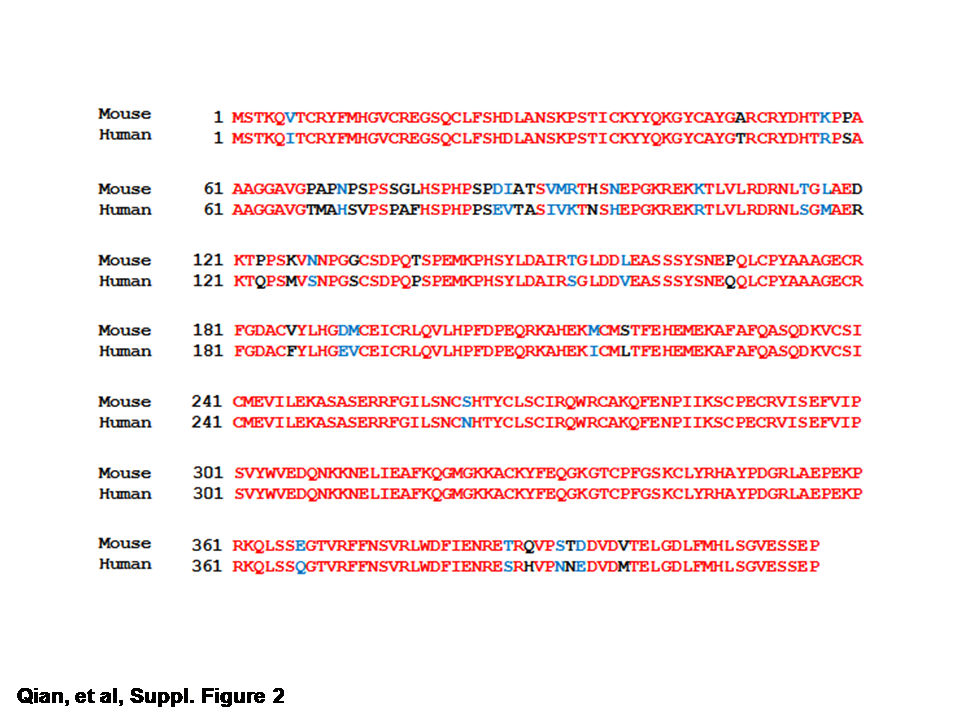

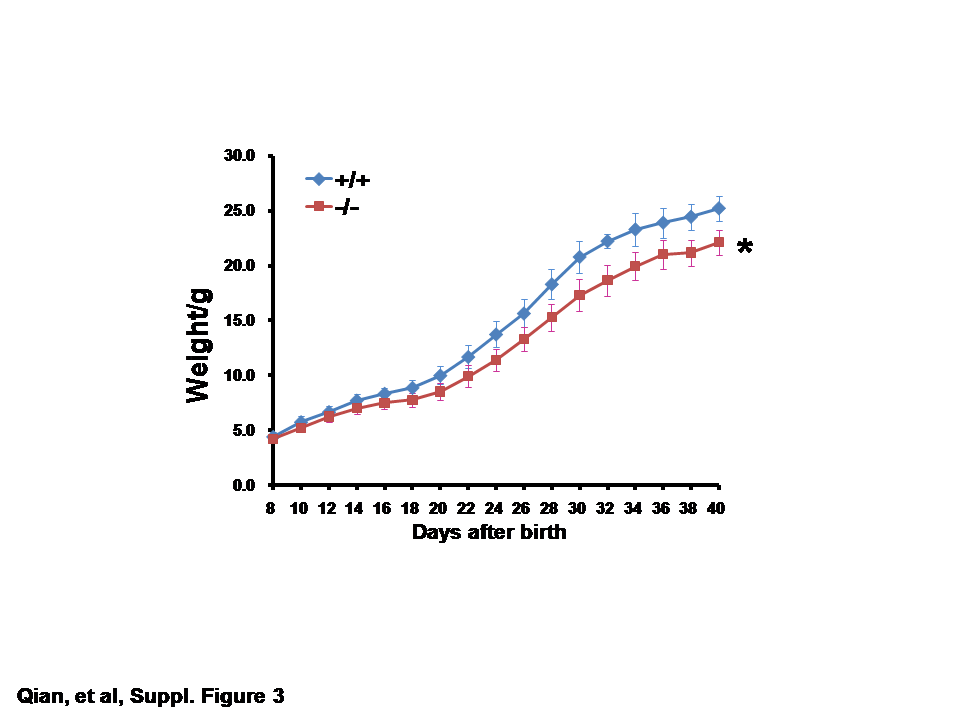

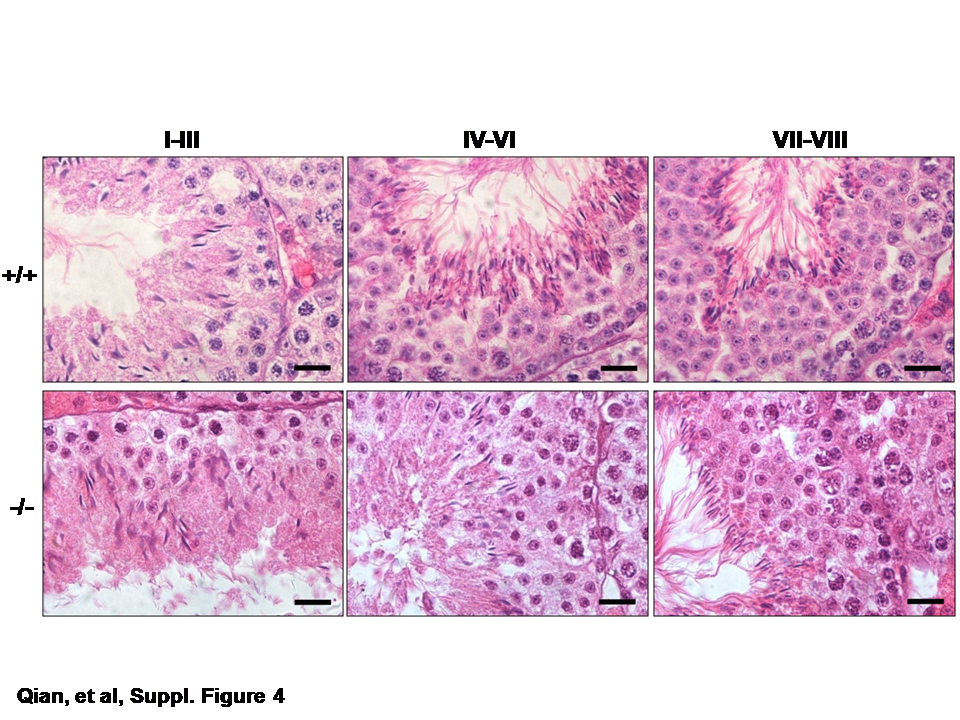

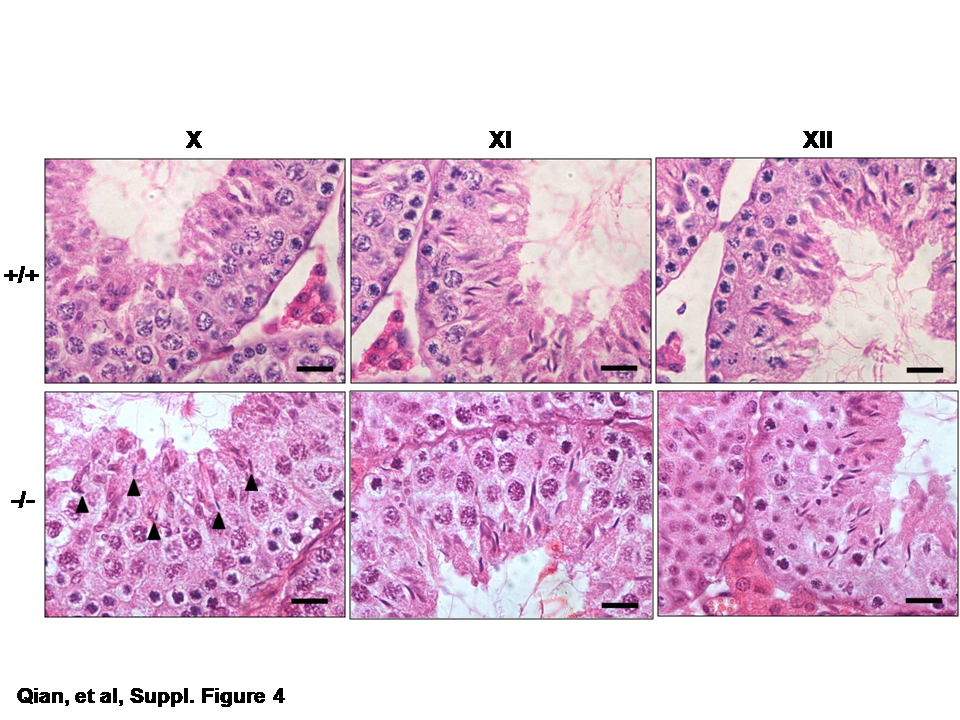

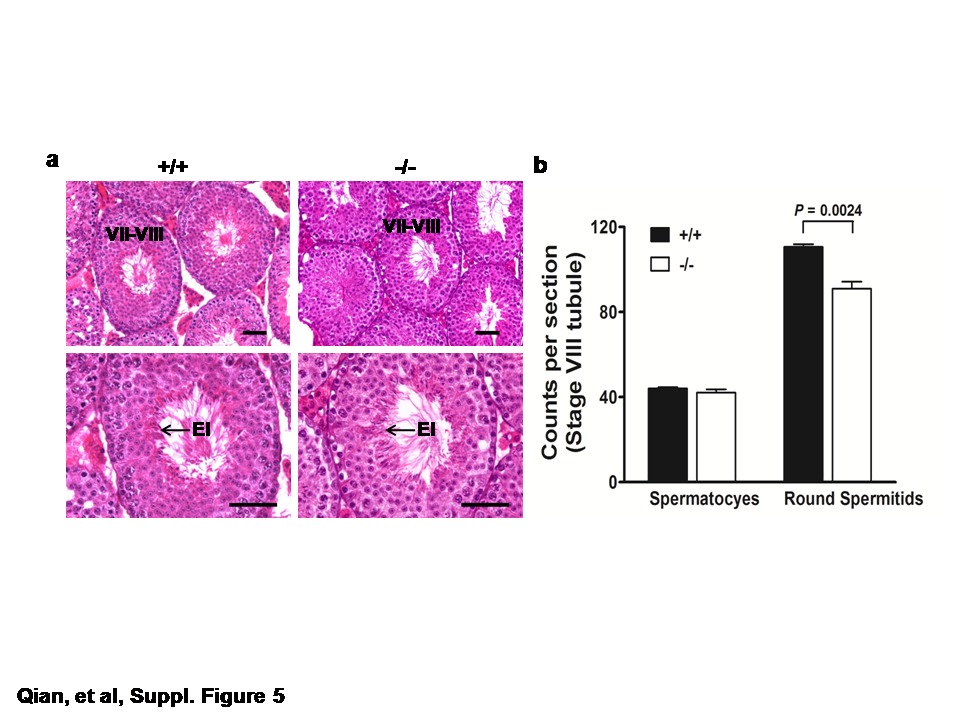

Supplement: Supplementary Information [file srep39318-s1.doc]
